# Safety and effectiveness of anticoagulation with non-vitamin K antagonist oral anticoagulants and warfarin in patients on tuberculosis treatment

**DOI:** 10.1038/s41598-023-29185-9

**Published:** 2023-02-04

**Authors:** Hyun-Jung Lee, Hyung-Kwan Kim, Bong-Seong Kim, Kyung-Do Han, Chan Soon Park, Tae-Min Rhee, Jun-Bean Park, Heesun Lee, Yong-Jin Kim

**Affiliations:** 1 Section of Cardiovascular Imaging, Division of Cardiology, Department of Internal Medicine, Cardiovascular Center, Seoul National University Hospital, Seoul National University College of Medicine, 101, Daehak-ro, Jongno-gu, Seoul, 03080 Korea; 2 Department of Statistics and Actuarial Science, Soongsil University, 369, Sangdo-ro, Dongjak-gu, Seoul, Korea; 3 Division of Cardiology, Department of Internal Medicine, Seoul National University Hospital Healthcare System Gangnam Center, 152, Teheran-ro, Gangnam-gu, Seoul, Korea

**Keywords:** Cardiology, Tuberculosis, Atrial fibrillation, Thromboembolism

## Abstract

Anti-tuberculosis treatment can cause significant drug-drug interaction and interfere with effective anticoagulation. However, there is a lack of evidence and conflicting data on the optimal oral anticoagulation in patients treated for tuberculosis. We investigated the safety and effectiveness of anticoagulation with non-vitamin K antagonist oral anticoagulants (NOACs) and warfarin in patients on anti-tuberculosis treatment. Patients on concomitant oral anticoagulation and anti-tuberculosis treatment including rifampin were identified from the Korean nationwide healthcare database. Subjects were censored at discontinuation of either anticoagulation or rifampin. The outcomes of interest were major bleeding, death, and ischemic stroke. A total 2090 patients (1153 on warfarin, 937 on NOAC) were included. NOAC users, compared to warfarin users, were older, had a lower prevalence of hypertension, heart failure, ischemic stroke, and aspirin use and a higher prevalence of cancer, with no significant differences in CHA_2_DS_2_-VASc or HAS-BLED scores. There were 18 major bleeding events, 106 deaths, and 50 stroke events during a mean follow-up of 2.9 months. After multivariable adjustment, the use of NOAC was associated with a lower risk of incident ischemic stroke (HR 0.51, 95% CI 0.27–0.94), while there was no significant difference in risk for major bleeding or death compared with warfarin. These results suggest that NOACs have better effectiveness for stroke prevention and similar safety compared with warfarin in patients on concomitant anti-tuberculosis treatment. This is the first study assessing the safety and effectiveness of NOACs compared to warfarin in this clinical scenario.

## Introduction

Tuberculosis (TB), caused by the bacillus *Mycobacterium tuberculosis* through air-borne transmission, remains a major cause of morbidity and mortality globally with approximately a quarter of the worldwide population affected^1^. Individuals with TB are categorized into active TB disease and latent TB infection. The standard treatment for people with drug-susceptible active TB disease is a 6-month regimen including four first-line drugs: isoniazid, rifampin, ethambutol and pyrazinamide^2^. Of the anti-TB medication, rifampin plays an essential role but is also the main cause of drug-drug interactions.

Patients with tuberculosis have increased risk of venous thromboembolism^3,4^, and can have comorbidities including atrial fibrillation and stroke, which require administration of anticoagulation. However, there is a lack of evidence and conflicting data on the optimal oral anticoagulation strategy in patients being treated for tuberculosis. Rifampin is a potent inducer of multiple pathways affecting drug metabolism including the hepatic cytochrome P450 (CYP) and P-glycoprotein (P-gp) efflux transporter system^5^. Warfarin is mainly metabolized in the liver via the CYP 2C9, 1A2, and 3A4^6^, and induction of the CYP enzymes leads to a significant decrease (about 15–74%) in warfarin area under the concentration–time curve (AUC)^7^. A three- to five-fold increase in warfarin dosage is usually required to attain sufficient warfarin levels measured by prothrombin time international normalized ratio (INR) testing^7,8^. The non-vitamin K antagonist oral anticoagulants (NOACs) are substrates of the either or both the CYP3A4 and P-gp system^9^, and induction of these systems by rifampin can also lead to reduced exposure to NOACs. Previous pharmacokinetic studies on co-administration of NOACs with rifampin reported that the AUC of dabigatran, apixaban, and rivaroxaban decreased 50–67%, while that of edoxaban decreased 35% but with compensatory increase of active metabolites^7,10–12^.

Thus, recent guidelines state that dabigatran, apixaban, and rivaroxaban are contraindicated while edoxaban may be used with caution when co-administered with rifampin, due to reduced NOAC plasma levels^13^. On the other hand, the risk of major bleeding was reported to be increased with the concurrent use of rifampin and NOACs in a retrospective cohort^14^, contrary to what may be expected from reduced NOAC levels. However, up to now, there have been no clinical studies focusing on the safety and effectiveness of NOACs compared to warfarin when used concomitantly with anti-TB medication^15^. In this study, we analyzed a nationwide cohort of patients on oral anticoagulation to compare the safety and effectiveness of NOACs and warfarin when co-administered with rifampin.

## Results

### Baseline characteristics of the study population

**Figure 1:**
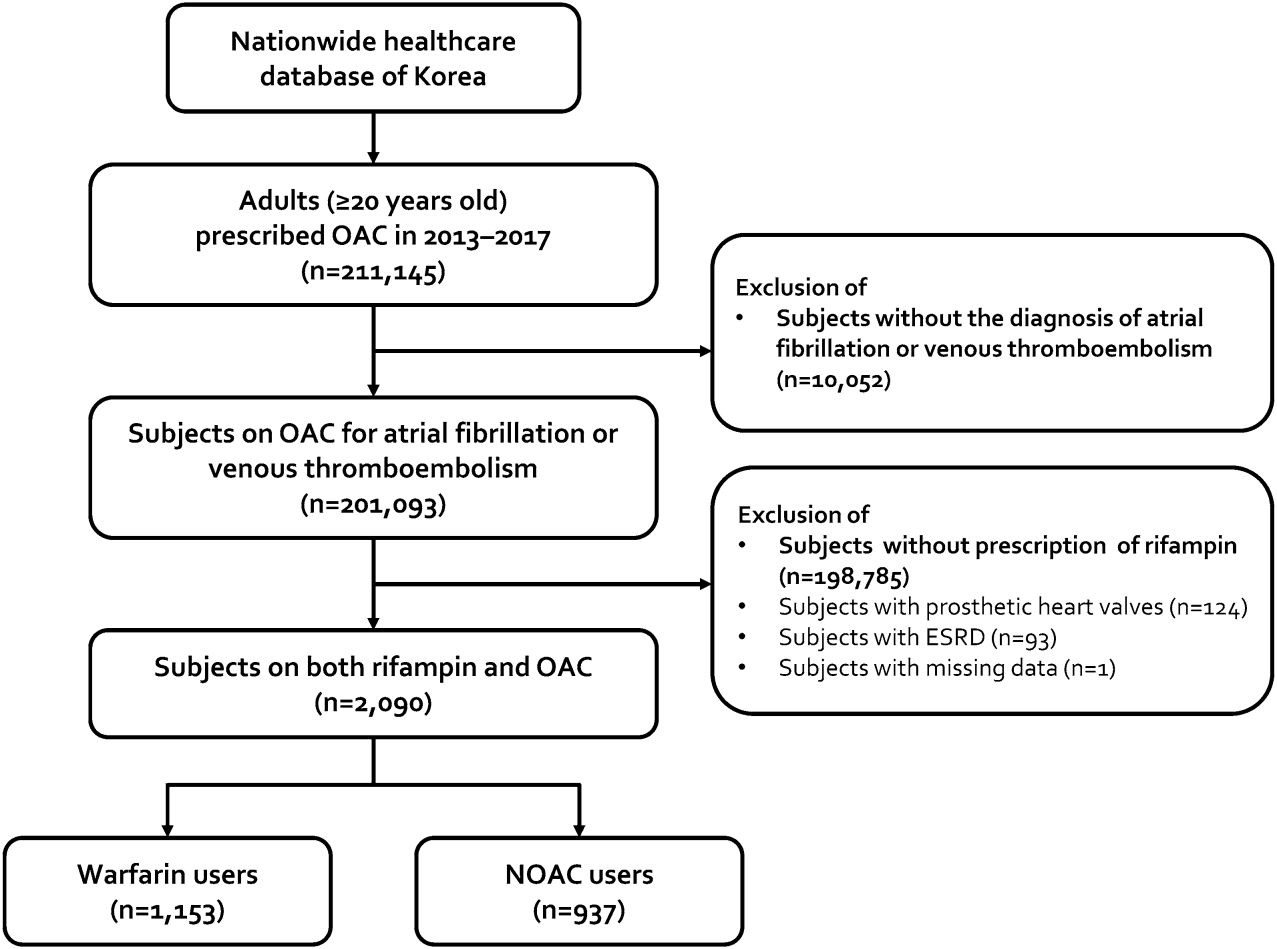
Study inclusion flow. *OAC* oral anticoagulant, *NOAC* non-vitamin K antagonist oral anticoagulant.

A total of 2090 subjects on concomitant oral anticoagulation and rifampin were included in the study, including 1153 patients on warfarin, and 937 patients on NOACs (Fig. 1). Indication for anticoagulation was atrial fibrillation in 1290 (61.7%), and venous thromboembolism in 800 (38.3%). The proportion of patients on NOAC versus warfarin increased over the years (Table 1). The proportion of atrial fibrillation as the indication for anticoagulation was higher in patients on warfarin. Patients on NOACs were on average 3 years older, had a lower prevalence of hypertension, heart failure, ischemic stroke, and aspirin use, higher prevalence of cancer, and less concomitant use of aspirin, compared to patients on warfarin. Meanwhile, while there were no significant differences in CHA_2_DS_2_-VASc or HAS-BLED scores, and history of intracranial hemorrhage or gastrointestinal bleeding between NOAC and warfarin users.

**Table 1:** Baseline characteristics of the study population.

	Total (n = 2090)	Warfarin users (n = 1153)	NOAC users (n = 937)	p-value
Year of enrollment				< 0.001
2013	406 (19.4)	364 (31.6)	42 (4.5)	
2014	397 (19.0)	302 (26.2)	95 (10.1)	
2015	369 (17.7)	212 (18.4)	157 (16.8)	
2016	442 (21.2)	157 (13.6)	285 (30.4)	
2017	476 (22.8)	118 (10.2)	358 (38.2)	
Indication for anticoagulation				
Atrial fibrillation	1290 (61.7)	805 (69.8)	485 (51.8)	< 0.001
Venous thromboembolism	800 (38.3)	348 (30.2)	452 (48.2)	< 0.001
Age	72.6 ± 12.3	71.4 ± 12.6	74.1 ± 11.7	< 0.001
Sex, male	1162 (55.6)	656 (56.9)	506 (54.0)	0.186
Low income (lowest 25%)	556 (26.6)	324 (28.1)	232 (24.8)	0.086
CHA_2_DS_2_-VASc score	4.0 ± 2.0	4.0 ± 2.0	4.1 ± 2.0	0.432
HAS-BLED score	2.5 ± 1.2	2.6 ± 1.2	2.5 ± 1.2	0.278
Comorbidities				
Hypertension	1562 (74.7)	883 (76.6)	679 (72.5)	0.031
Diabetes mellitus	650 (31.1)	379 (32.9)	271 (28.9)	0.053
Dyslipidemia	798 (38.2)	455 (39.5)	343 (36.6)	0.181
Myocardial infarction	108 (5.2)	66 (5.7)	42 (4.5)	0.202
Heart failure	807 (38.6)	486 (42.2)	321 (34.3)	< 0.001
Ischemic stroke	402 (19.2)	250 (21.7)	152 (16.2)	0.002
Intracranial hemorrhage	42 (2.0)	20 (1.7)	22 (2.4)	0.320
Gastrointestinal bleeding	49 (2.3)	28 (2.4)	21 (2.2)	0.778
Chronic kidney disease	186 (8.9)	114 (9.9)	72 (7.7)	0.079
Cancer	242 (11.6)	105 (9.1)	137 (14.6)	< 0.001
Concurrent medication				
Aspirin	221 (10.6)	150 (13.0)	71 (7.6)	< 0.001
P2Y12 inhibitor	100 (4.8)	64 (5.6)	36 (3.8)	0.0687
NSAID	162 (7.8)	95 (8.2)	67 (7.2)	0.3545

*NSAID* non-steroidal anti-inflammatory drug.

### Risk of outcomes associated with use of warfarin or NOACs

The mean follow-up duration was 2.9 ± 3.0 months. Overall, the incidence of major bleeding events during concomitant anticoagulation and anti-TB medication was low. Major bleeding events occurred in 6 of warfarin users (0.52%) and 12 of NOAC users (1.28%). Death occurred in 48 of warfarin users (4.2%) and 58 of NOAC users (6.2%). Ischemic stroke occurred in 35 patients on warfarin (3.0%) and 15 patients on NOAC (1.6%); when confined to the subgroup of patients on anticoagulation for atrial fibrillation (n = 1290), ischemic stroke occurred in 34 of 805 warfarin users (4.2%) and 12 of NOAC users (2.5%).

**Figure 2:**
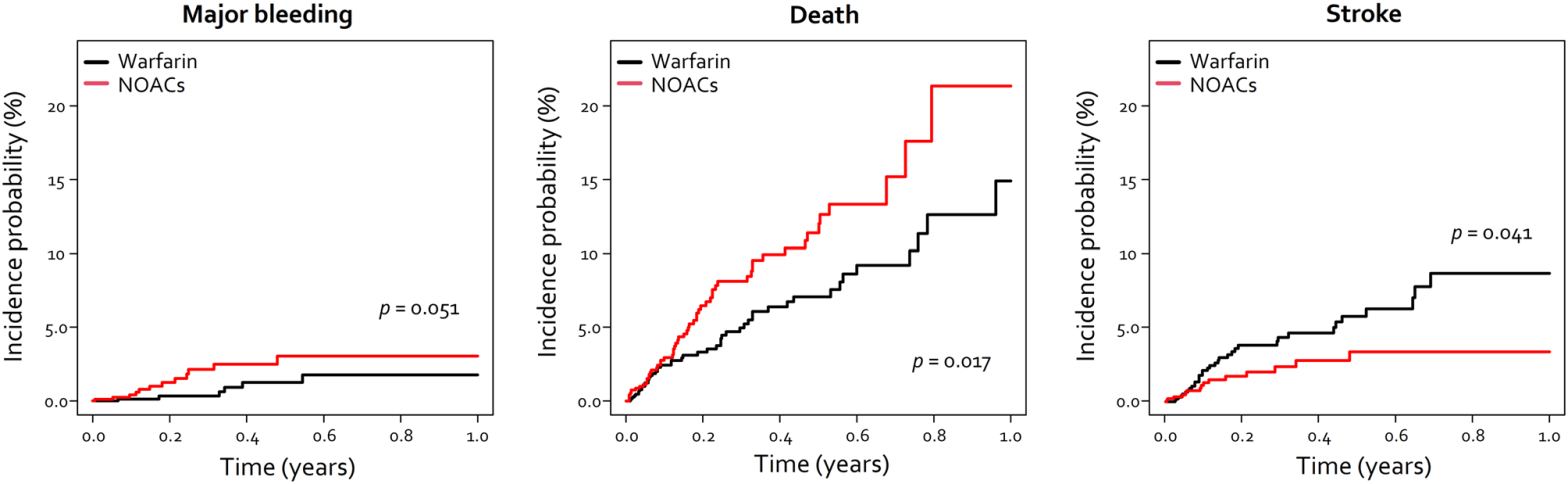
Crude incidence of outcomes in warfarin and NOAC users by Kaplan–Meier analysis. *NOAC* non-vitamin K antagonist oral anticoagulant.

There was a tendency for higher incidence of major bleeding and death and lower incidence of stroke in NOAC users compared to warfarin users by unadjusted Kaplan–Meier survival analysis (Fig. 2). The use of NOAC was associated with higher risk of death and lower risk of stroke compared to use of warfarin in unadjusted univariable Cox regression analysis (Table 2). After multivariable adjustment for possible confounding factors, the use of NOAC was associated with a lower risk of incident ischemic stroke (HR 0.51, 95% confidence interval [CI] 0.27–0.94, p = 0.030); meanwhile, the risk for major bleeding or death was no longer significantly different compared to use of warfarin (Table 2). The tendency for lower risk of stroke with use of warfarin compared to use of NOAC was consistent when confined to the patients being anticoagulated for atrial fibrillation, though not achieving significance (p = 0.086).

**Table 2:** Safety and effectiveness of anticoagulation in warfarin and NOAC users.

	N	Event	IR	Univariable Cox analysis		Multivariable Cox analysis^a^	
				HR (95% CI)	p-value	HR (95% CI)	p-value
Major bleeding							
Warfarin	1153	6	2.11	1 (ref)		1 (ref)	
NOAC	937	12	5.67	2.56 (0.96–6.84)	0.060	2.15 (0.77–5.98)	0.144
Death							
Warfarin	1153	48	16.8	1 (ref)		1 (ref)	
NOAC	937	58	27.1	1.59 (1.08–2.34)	0.018	1.44 (0.98–2.13)	0.065
Stroke							
Warfarin	1153	35	12.5	1 (ref)		1 (ref)	
NOAC	937	15	7.12	0.54 (0.29–0.98)	0.044	0.51 (0.27–0.94)	0.030
Stroke in subjects with atrial fibrillation							
Warfarin	805	33	17.8	1 (ref)		1 (ref)	
NOAC	485	12	11.0	0.58 (0.30–1.12)	0.105	0.55 (0.28–1.09)	0.086

*NOAC* non-vitamin K antagonist oral anticoagulant.

^a^Adjusted for age, sex, CHA_2_DS_2_-VASc score, history of intracranial hemorrhage, history of gastrointestinal bleeding, chronic kidney disease, cancer, and concurrent antiplatelet usage.

### Reduced dose analysis

Among NOAC users, 52.0% (n = 487) were prescribed reduced dose of NOACs. Supplemental Table 1 shows the hazard ratios for the study outcomes for reduced and standard dose of NOACs compared to warfarin. The trend of lower risk for stroke and higher risk for death and bleeding associated with NOACs were consistent regardless of dose regimen, and after multivariable adjustment, only the association between reduced dose of NOACs and increased risk of death was significant. Supplemental Table 2 shows the hazard ratios for the study outcomes for reduced dose of NOACs compared to standard dose of NOACs. After multivariable adjustment, no significant difference was observed between reduced dose of NOACs and standard dose of NOACs for all study outcomes.

### Subgroup analysis

**Figure 3:**
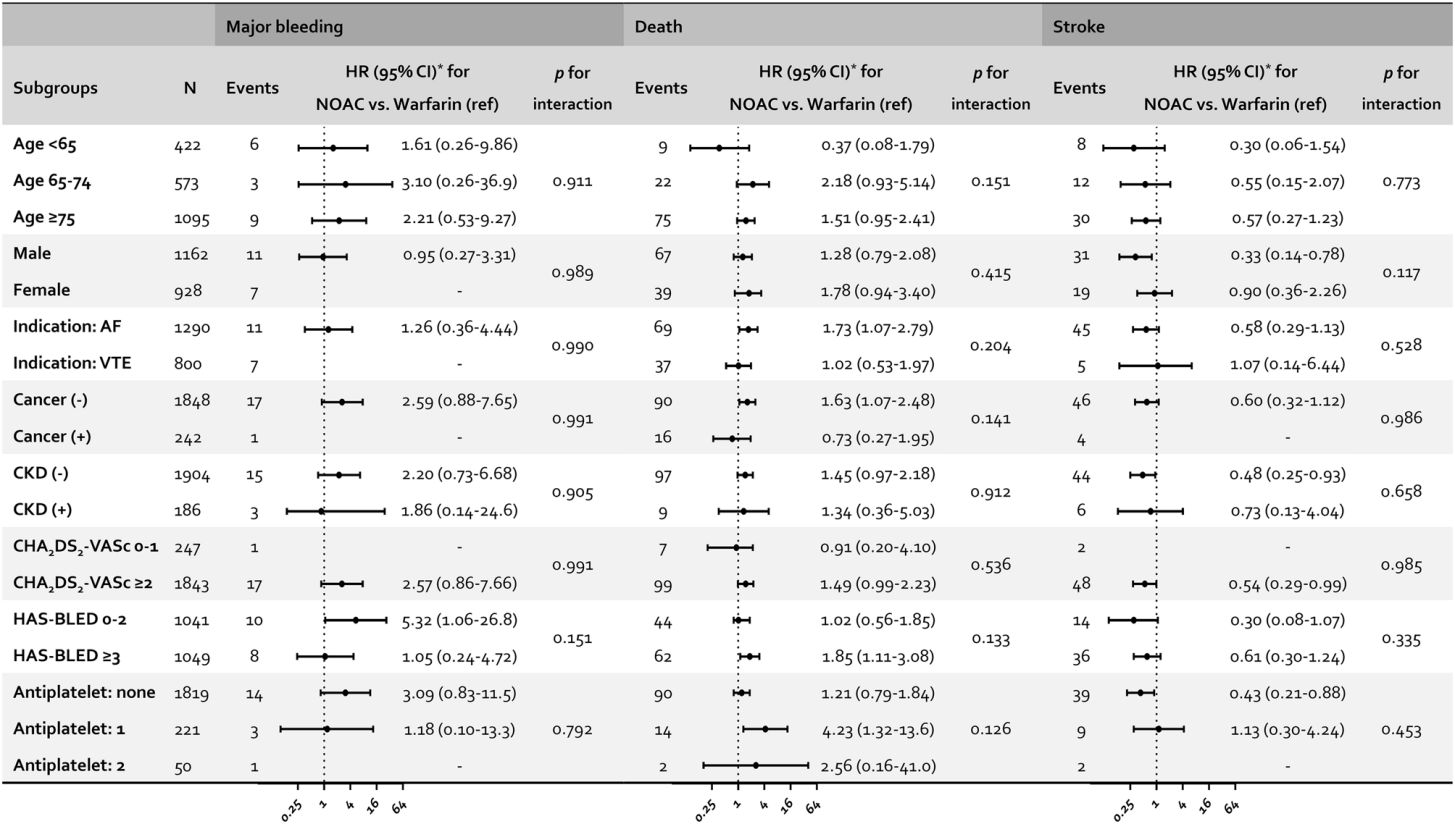
Subgroup analyses of risk for outcomes in warfarin and NOAC users. Adjusted for age, sex, CHA_2_DS_2_-VASc score, history of intracranial hemorrhage, history of gastrointestinal bleeding, chronic kidney disease, cancer, and concurrent antiplatelet usage. *HR* hazard ratio, *NOAC* non-vitamin K antagonist oral anticoagulant, *AF* atrial fibrillation, *VTE* venous thromboembolism, *CKD* chronic kidney disease.

Subgroup analyses stratified by age, sex, indication for OAC use, history of cancer or chronic kidney disease, stroke risk according to the CHA_2_DS_2_-VASc score, and bleeding risk according to the HAS-BLED score and number of concomitant antiplatelet agents were performed, and no significant subgroup difference detected in the treatment effect of NOAC versus warfarin (all p-for-interaction > 0.100) (Fig. 3). However, this may be due to the lack of power. To note, the incidence of major bleeding events was similar in patients with HAS-BLED score 0–2 and ≥ 3, and there was a tendency for higher bleeding risk associated with NOACs compared to warfarin in patients with low HAS-BLED scores. Most of the stroke events occurred in patients with CHA_2_DS_2_-VASc scores ≥ 2, and NOAC was associated with significantly lower risk of stroke in patients with high CHA_2_DS_2_-VASc scores compared to warfarin.

## Discussion

To the best of our knowledge, this is the first and largest study to comprehensively compare the safety and effectiveness of NOACs with warfarin in patients during concomitant TB medication. Our key findings are as following: (1) major bleeding events are rare and stroke events mostly occur in patients with high CHA_2_DS_2_-VASc scores; (2) NOACs were associated with better effectiveness for stroke prevention and similar safety regarding major bleeding and death on multivariate analysis; (3) stratified analyses according to NOAC dose and subgroups showed results overall consistent with the main analysis.

TB is a global health concern, being the 13th leading cause of death worldwide and the leading cause of death from infection in 2019^1^. TB is prevalent in low and middle income countries with widespread poverty, undernutrition, human immunodeficiency virus infection, smoking and diabetes. Korea still has a high burden of TB, with the highest incidence of TB (49 cases per 100,000 population) and the third highest mortality from TB (3.8 cases per 100,000 population) of the 38 member countries of the Organization for Economic Cooperation and Development^1^. On the other hand, the prevalence of atrial fibrillation continues to increase rapidly with population aging; the prevalence of atrial fibrillation in Korea was 1.53% in 2015 and continues to increase over time^16^. The incidence of venous thromboembolism is also on the rise^17^. Meanwhile, TB infection is associated with increased thrombogenicity^3,4^. Thus, not infrequently, clinicians encounter cases in which patients on anticoagulation start anti-TB medication or vice-versa; however, there is a lack of evidence on the best oral anticoagulation strategy during concomitant anti-TB medication. Some clinicians make the choice of switching to warfarin and monitoring INR levels in these patients, instead of maintaining NOACs.

The use of rifampin causes induction of the hepatic CYP and P-gp enzymes related to drug metabolism and subsequently leads to a reduction in drug exposure for both warfarin and NOACs according to pharmacokinetic studies^7,10–12^. The dose of warfarin is usually increased during concomitant anti-TB medication with INR monitoring to achieve target levels^7,8^, while there is no method to monitor NOAC activity and no consensus on increasing NOAC dose during concomitant anti-TB medication. The latest guideline states that edoxaban may be used while the other NOACs are contraindicated due to reduced plasma levels^13^. One can presume that lower plasma levels of NOACs would result in more stroke events but less bleeding; however, a retrospective cohort study including 1151 patients on concomitant rifampin and NOACs from the Taiwan National Health Insurance database reported that in patients taking NOACs for atrial fibrillation, the incidence of major bleeding increased with the concurrent use of rifampin^14^.

In the current retrospective cohort, we found an insignificant tendency for increased major bleeding and death, and a significant decrease in stroke events associated with NOACs compared to warfarin. Presuming that the dose of warfarin was increased while that of NOAC was not during concomitant anti-TB medication, we expected that the incidence of bleeding events would be higher in warfarin users. However, results were contrary to our expectation, suggesting that anticoagulation effects of NOAC remain stronger than that of warfarin. This suggests that there may be other drug–drug interactions in addition to those currently established while on concurrent anticoagulation and anti-TB medication, translating into unpredictable drug exposure levels and outcomes. This warrants further research for underlying in vivo mechanisms.

Ischemic stroke events mostly occurred in patients with high CHA2DS2-VASc scores (≥ 2), and NOACs were associated with significantly lower risk of stroke compared to warfarin. While there was a tendency for increased bleeding with NOACs compared to warfarin, the actual incidence of major bleeding was low and the difference was insignificant after multivariable adjustment. Also, the bleeding risk related to NOACs was not especially higher in those with higher bleeding risk (HAS-BLED scores ≥ 3). These results suggest that clinicians should continue anticoagulation in patients at high risk of stroke, preferably with NOACs than warfarin, during treatment for TB.

### Strengths and limitations

A main strength of this study is that it included all Korean patients on a nationwide basis prescribed both oral anticoagulants and anti-TB medication and is thus free from referral bias; also, complete outcomes without loss of follow-up could be obtained from the database. Nevertheless, there are some limitations that are worthy of being mentioned. First, as we limited the study period to the duration of concomitant oral anticoagulation and anti-TB medication, the mean follow-up was around 3 months and the number of outcome events was not high, resulting in decreased statistical power. This was inevitable considering that the standard anti-TB regimen usually lasts only 6 months. Second, we also planned to compare outcomes between the individual NOACs and verify whether edoxaban was associated with better outcome as presumed in the latest guideline^13^, but the study lacked statistical power to undergo these analyses. Third, due to the small number of major bleeding events, there may be a small positive association between NOAC use and increased bleeding risk which was missed in multivariate analysis related to the lack of power. However, this also suggests that the risk of major bleeding is practically low during the short period of concomitant anticoagulation and anti-TB treatment, and the difference in risk between use of NOAC and warfarin negligible. Fourth, as the INR levels were not available in the database, we could not assess the time in therapeutic range for warfarin users. Last, due to the retrospective observational nature of the study, our study cannot provide causal relationships and there should be caution in interpreting the results. Despite these limitations, we believe that this study is of practical value, considering that randomized clinical trials regarding this issue are unlikely to be performed in this special population.

## Conclusions

In a nationwide cohort of patients on concomitant anti-TB medication and oral anticoagulation, NOACs showed better effectiveness for stroke prevention and no significant difference in safety regarding major bleeding and death compared with warfarin. These results suggest that clinicians should continue anticoagulation preferably with NOACs than warfarin during treatment for TB in patients at high risk of stroke, rather than switching to warfarin and monitoring INR levels.

## Methods

### Study population

The study cohort was derived from the Korean National Health Insurance Service claims database covering the entire Korean population, the details of which have been described previously^18,19^. De-identified datasets are provided to approved researchers at the National Health Insurance Sharing Service (nhiss.nhis.or.kr). The current study complied with the Declaration of Helsinki, and was approved by the Institutional Review Board of Seoul National University Hospital (E-2208-109-1351). Informed consent was waived by the Institutional Review Board of Seoul National University Hospital due to the retrospective nature of the study and anonymized database.

Adults (age ≥ 20 years) who were prescribed oral anticoagulants for the clinical indications of non-valvular atrial fibrillation or venous thromboembolism between January 2013 and December 2017 were identified. This population was provided after 50% random sampling from the database, according to regulations of the Korean National Health Insurance Service. Oral anticoagulants included warfarin and the NOACs, i.e. rivaroxaban, dabigatran, apixaban, and edoxaban. The starting year was set as 2013, the year NOACs were approved for use in Korea. From this population, we included patients with simultaneous prescription of rifampin (Fig. 1). Rifampin was selected to identify patients on anti-TB treatment, as it is an integral part throughout the standard 6-month anti-TB regimen and is the main cause of drug-drug interaction. We excluded patients with prosthetic heart valves, end-stage renal disease, or missing data. The index date was the first date of concomitant prescription of oral anticoagulants and rifampin. Subjects were censored at the discontinuation of either anticoagulation or rifampin, or at 1 year to reduce confounding effects related to outliers. Follow-up was until the occurrence of the outcomes, death, censoring, or the end of the study (December 31, 2018).

### Covariates and study outcomes

Age, sex, and income level were obtained from the database. Comorbidities were assessed for 1 year before the index date. Diseases were defined using diagnostic codes, inpatient and outpatient hospital visits, and prescription codes (Supplemental Table 3)^19,20^. Concomitant antiplatelet or nonsteroidal anti-inflammatory drug (NSAID) use of at least 1 month or more during the study period was identified. The HAS-BLED score was calculated as a measure of bleeding risk, by assessing the presence of hypertension (1 point), abnormal renal function (1 point; end-stage renal disease, chronic kidney disease, kidney transplantation), abnormal liver function (1 point; liver cirrhosis, liver disease), stroke (1 point; ischemic stroke), bleeding (1 point; previous hospitalization for gastrointestinal bleeding, peptic ulcer), old age (1 point; age > 65 years), heavy alcohol drinking (1 point; > 8 times/week), and antiplatelet or NSAID use (1 point)^21^. The CHA_2_DS_2_-VASc score was calculated as a measure of stroke risk, by assessing the presence of congestive heart failure (1 point), hypertension (1 point), old age (2 points if ≥ 75 years; 1 point if ≥ 65 years), diabetes mellitus (1 point), prior stroke or transient ischemic attack or systemic embolism (2 points), vascular disease (1 point; myocardial infarction or peripheral artery disease), and female sex (1 point)^22^.

The safety outcomes were hospitalization for major bleeding and all-causes death. Major bleeding was defined as intracranial hemorrhage, gastrointestinal bleeding, respiratory tract bleeding, and internal bleeding such as hemothorax, hemoperitoneum, and hemopericardium. The effectiveness outcome was hospitalization for ischemic stroke. The outcome of ischemic stroke was assessed in the total study population and in the subgroup of patients with atrial fibrillation.

### Statistical analysis

Categorical data are presented as numbers (%), and continuous data are presented as mean ± standard deviation. Characteristics were compared between the groups using the t-test or the chi-square test. Incidence rates were calculated by dividing the number of events by the total follow-up period (per 1000 person-years). The risks of the outcomes associated with warfarin or NOAC use were assessed with Cox regression analysis, and the hazard ratios (HR) and corresponding 95% CI were estimated. Cumulative incidence curves for the outcomes were drawn by the Kaplan–Meier method and compared with the log-rank test. Multivariate models were adjusted for age, sex, CHA_2_DS_2_-VASc score, history of intracranial hemorrhage, history of gastrointestinal bleeding, chronic kidney disease, cancer, and concurrent antiplatelet usage. We also performed separate analyses for the outcomes stratified by the NOAC dose (i.e., standard and reduced). Reduced doses of NOACs were defined as 15 or 10 mg rivaroxaban once daily, 110 mg dabigatran twice daily, 2.5 mg apixaban twice daily, and 30 mg edoxaban once daily, while regular doses of NOACs were defined as 20 mg rivaroxaban once daily, 150 mg dabigatran twice daily, 5 mg apixaban twice daily, and 60 mg edoxaban once daily. Pre-specified subgroup analyses were performed according to age, sex, indication for anticoagulation, cancer, chronic kidney disease, CHA_2_DS_2_-VASc score and HAS-BLED score, and number of antiplatelet agents. P-values < 0.05 were considered statistically significant. SAS version 9.4 (SAS Institute, Cary, NC, USA) was used for all statistical analyses.

## Supplementary Information

Supplementary Tables.

## Data Availability

The dataset generated and analyzed in the current study are available in the Korean National Health Insurance Sharing Service database (https://nhiss.nhis.or.kr) and specifications required for the dataset are available from the corresponding author on reasonable request.
