# Supplementary material for: Safety and effectiveness of anticoagulation with non-vitamin K antagonist oral anticoagulants and warfarin in patients on tuberculosis treatment
Source: Sci Rep. 2023 Feb 4;13:2060. doi: 10.1038/s41598-023-29185-9 (PMC9899262; doi:10.1038/s41598-023-29185-9)
Supplement: Supplementary file 1 — Supplementary Tables. [file 41598_2023_29185_MOESM1_ESM.docx]

**SUPPLEMENTAL MATERIALS**

**Safety and effectiveness of anticoagulation with non-vitamin K antagonists and warfarin in patients on tuberculosis treatment**

Hyun-Jung Lee, MD^1^, Hyung-Kwan Kim, MD, PhD^1,*^, Bong-Seong Kim^2^,

Kyung-Do Han, PhD^2^, Chan Soon Park, MD^1^, Tae-Min Rhee, MD^1^,

Jun-Bean Park, MD, PhD^1^, Heesun Lee, MD, PhD^3^, Yong-Jin Kim, MD, PhD^1^

^1^Division of Cardiology, Department of Internal Medicine, Cardiovascular Center, Seoul National University Hospital, Seoul National University College of Medicine, 101, Daehak-ro, Jongno-gu, Seoul, Korea

^2^Department of Statistics and Actuarial Science, Soongsil University, 369, Sangdo-ro, Dongjak-gu, Seoul, Korea

^3^Division of Cardiology, Department of Internal Medicine, Seoul National University Hospital Healthcare System Gangnam Center, 152, Teheran-ro, Gangnam-gu, Seoul, Korea

**Table of Contents**

1. Supplemental Tables 1–3

**SUPPLEMENTAL TABLES**

**Supplemental Table 1. Safety and effectiveness of anticoagulation in warfarin and NOAC users according to NOAC dosage**

|  | **N** | **Event** | **IR** | **Univariable Cox analysis** | | **Multivariable Cox analysis^*^** | |
| --- | --- | --- | --- | --- | --- | --- | --- |
|  |  |  |  | HR (95% CI) | p-value | HR (95% CI) | p-value |
| **Major bleeding** |  |  |  |  |  |  |  |
| Warfarin | 1153 | 6 | 2.11 | 1 (ref) |  | 1 (ref) |  |
| Reduced dose NOAC | 487 | 7 | 6.70 | 3.08 (1.03-9.16) | 0.044 | 1.87 (0.56-5.99) | 0.289 |
| Standard dose NOAC | 450 | 5 | 4.67 | 2.08 (0.63-6.82) | 0.228 | 2.63 (0.75-9.26) | 0.132 |
| **Death** |  |  |  |  |  |  |  |
| Warfarin | 1153 | 48 | 16.8 | 1 (ref) |  | 1 (ref) |  |
| Reduced dose NOAC | 487 | 37 | 35.1 | 2.05 (1.33-3.16) | 0.001 | 1.73 (1.12-2.67) | 0.014 |
| Standard dose NOAC | 450 | 21 | 19.4 | 1.14 (0.68-1.91) | 0.619 | 1.12 (0.66-1.88) | 0.673 |
| **Stroke** |  |  |  |  |  |  |  |
| Warfarin | 1153 | 35 | 12.5 | 1 (ref) |  | 1 (ref) |  |
| Reduced dose NOAC | 487 | 9 | 8.65 | 0.65 (0.31-1.36) | 0.252 | 0.59 (0.28-1.24) | 0.161 |
| Standard dose NOAC | 450 | 6 | 5.63 | 0.42 (0.18-1.01) | 0.052 | 0.42 (0.18-1.01) | 0.053 |
| **Stroke in subjects with atrial fibrillation** | | | |  |  |  |  |
| Warfarin | 805 | 33 | 17.8 | 1 (ref) |  | 1 (ref) |  |
| Reduced dose NOAC | 308 | 9 | 13.0 | 0.69 (0.33-1.45) | 0.327 | 0.69 (0.32-1.47) | 0.338 |
| Standard dose NOAC | 177 | 3 | 7.56 | 0.39 (0.12-1.27) | 0.117 | 0.35 (0.10-1.15) | 0.083 |

^*^Adjusted for age, sex, CHA_2_DS_2_-VASc score, history of intracranial hemorrhage, history of gastrointestinal bleeding, chronic kidney disease, cancer, and concurrent antiplatelet usage.

NOAC, Non-vitamin K antagonist oral anticoagulant.

**Supplemental Table 2. Safety and effectiveness of anticoagulation in NOAC users according to NOAC dosage**

|  | **N** | **Event** | **IR** | **Univariable Cox analysis** | | **Multivariable Cox analysis^*^** | |
| --- | --- | --- | --- | --- | --- | --- | --- |
|  |  |  |  | HR (95% CI) | p-value | HR (95% CI) | p-value |
| **Major bleeding** |  |  |  |  |  |  |  |
| Reduced dose NOAC | 487 | 7 | 6.70 | 1.48 (0.47-4.67) | 0.503 | 0.88 (0.23-3.41) | 0.851 |
| Standard dose NOAC | 450 | 5 | 4.67 | 1 (ref) |  | 1 (ref) |  |
| **Death** |  |  |  |  |  |  |  |
| Reduced dose NOAC | 487 | 37 | 35.1 | 1.81 (1.06-3.10) | 0.030 | 1.50 (0.86-2.60) | 0.151 |
| Standard dose NOAC | 450 | 21 | 19.4 | 1 (ref) |  | 1 (ref) |  |
| **Stroke** |  |  |  |  |  |  |  |
| Reduced dose NOAC | 487 | 9 | 8.65 | 1.55 (0.55-4.35) | 0.407 | 1.31 (0.44-3.89) | 0.626 |
| Standard dose NOAC | 450 | 6 | 5.63 | 1 (ref) |  | 1 (ref) |  |
| **Stroke in subjects with atrial fibrillation** | | | |  |  |  |  |
| Reduced dose NOAC | 308 | 9 | 13.0 | 1.80 (0.49-6.65) | 0.378 | 1.50 (0.39-5.82) | 0.557 |
| Standard dose NOAC | 177 | 3 | 7.56 | 1 (ref) |  | 1 (ref) |  |

^*^Adjusted for age, sex, CHA_2_DS_2_-VASc score, history of intracranial hemorrhage, history of gastrointestinal bleeding, chronic kidney disease, cancer, and concurrent antiplatelet usage.

NOAC, Non-vitamin K antagonist oral anticoagulant.

**Supplemental Table 3. Definitions of comorbidities and outcomes**

| **Variables** | **ICD-10 codes** | **Additional definitions** |
| --- | --- | --- |
| *Covariates^*^* |  |  |
| Atrial fibrillation | I48 | Admission ≥ 1 or outpatient clinic ≥ 1 |
| Venous thromboembolism | I26 (pulmonary thromboembolism), I80-82 (deep vein thrombosis) | Admission ≥ 1 or outpatient clinic ≥ 1 |
| Presence of prosthetic heart valve | Z95.2-95.4 | Admission ≥ 1 or outpatient clinic ≥ 1 |
| End-stage renal disease | N18.5, N18.9, N19, Z49, Z99.2 | 1) Dialysis ≥ 2 [Procedure codes: O7011-7020 (hemodialysis), O7071-O7075 (peritoneal dialysis)]  or 2) Registration for rare disease [V001 (hemodialysis), V003 (peritoneal dialysis)] |
| Hypertension | I10-I13, I15 | Admission ≥ 1 or outpatient clinic ≥ 2 Minimum 1 prescription of anti-hypertensive drug (thiazide, loop diuretics, aldosterone antagonist, alpha-/beta-blocker, calcium-channel blocker, angiotensin-converting enzyme inhibitor, angiotensin II receptor blocker) |
| Diabetes mellitus | E11-E14 | Admission ≥ 1 or outpatient clinic ≥ 2 Minimum 1 prescription of anti-diabetic drugs (sulfonylureas, metformin, meglitinides, thiazolidinediones, dipeptidyl peptidase-4 inhibitors, α-glucosidase inhibitors, SGLT2-inhibitor, GLP-1 agonist, or insulin) |
| Dyslipidemia | E78 | Admission ≥ 1 or outpatient clinic ≥ 2 Minimum 1 prescription of lipid-lowering medication (statin, ezetimibe, fenofibrate) |
| Myocardial infarction | I21, I22 | Admission ≥ 1 or outpatient clinic ≥ 1 |
| Heart failure | I50, I42.0, I11.0, I13.0, I13.2 | Admission ≥ 1 or outpatient clinic ≥ 1 |
| Ischemic stroke | I63, I64 | Admission ≥ 1 or outpatient clinic ≥ 1 |
| Intracranial hemorrhage | I63, I64 | Admission ≥ 1 or outpatient clinic ≥ 1 |
| Gastrointestinal bleeding | K25.0, K25.2, K25.4, K25.6, K26.0, K26.2, K26.4, K26.6, K27.0, K27.2, K27.4, K27.6, K28.0, K28.2, K28.4, K28.6, K29.0, K92.0-92.2, I85.0, K22.6, K55.2, K62.5, K63.3, K64.9 | Admission ≥ 1 or outpatient clinic ≥ 1 |
| Chronic kidney disease | N00-007, N11, I12, N18-19, Q61 | Admission ≥ 1 or outpatient clinic ≥ 1 |
| Cancer | C00-97 | Admission ≥ 1 or outpatient clinic ≥ 1 |
| *Endpoints* |  |  |
| Ischemic stroke | I63, I64 | Admission ≥ 1 with claims for brain CT or MRI |
| Major bleeding |  |  |
| 1. Intracranial hemorrhage | I60-62 | Primary diagnosis + Admission ≥ 1 |
| 1. Admission for GI bleeding | K25.0, K25.2, K25.4, K25.6, K26.0, K26.2, K26.4, K26.6, K27.0, K27.2, K27.4, K27.6, K28.0, K28.2, K28.4, K28.6, K29.0, K92.0-92.2, I85.0, K22.6, K55.2, K62.5, K63.3, K64.9 | Primary diagnosis + Admission ≥ 1 |
| 1. Admission for respiratory tract bleeding | R04 | Primary diagnosis + Admission ≥ 1 |
| 1. Admission for internal bleeding | J94.2 (hemothorax), K66.1 (hemoperitoneum), I31.2 (hemopericardium) | Primary diagnosis + Admission ≥ 1 |
| *Risk scores* | *Components* |  |
| HAS-BLED score^†^ | Hypertension (1 point); Abnormal renal function (1 point; end-stage renal disease, chronic kidney disease, kidney transplantation); Abnormal liver function (1 point; liver cirrhosis, liver disease); Stroke (1 point; ischemic stroke); Bleeding (1 point; previous hospitalization for GI bleeding, peptic ulcer); Elderly (1 point; age > 65 years); Alcohol (1 point; > 8 times/week); Antiplatelet or NSAID use (1 point) | |
| CHA_2_DS_2_-VASc score | Congestive heart failure (1 point); Hypertension (1 point); Age (2 points if ≥ 75 years; 1 point if ≥ 65 years); Diabetes mellitus (1 point); Prior stroke or transient ischemic attack or systemic embolism (2 points); Vascular disease (1 point; myocardial infarction or peripheral artery disease); Female sex (1 point) | |

*Covariates were defined by diagnoses during hospitalization or at outpatient clinic during the past 1 year.

^†^ Labile prothrombin time was not included in the score due to lack of data.

CT, computed tomography; GI, gastrointestinal; ICD-10, International Classification of Diseases 10^th^ revision from the World Health Organization; MRI, magnetic resonance imaging.
